# Accurate 24-h urine cystine quantification for patients on cystine-binding thiol drugs

**DOI:** 10.1007/s00240-022-01364-9

**Published:** 2022-10-06

**Authors:** Charles C. Mikel, David S. Goldfarb, Amy Ponte, Katherine Steigelman, Sergey Latyshev

**Affiliations:** 1 Nightmaker Science, LLC, 9924 Mesa Rim Road, Suite A, San Diego, CA 92121 USA; 2 Nephrology Section, New York Harbor VAMC, St. Vincent’s Hospital, New York, NY USA; 3 Travere Therapeutics, Inc., San Diego, CA USA

**Keywords:** Cystinuria, Kidney stones, Cystine-binding thiol drug, Liquid chromatography tandem mass spectrometry, Urinary cystine solubility

## Abstract

Cystinuria is a rare disorder resulting in development of recurrent kidney stones, adversely affecting patient quality of life. The goal of cystinuria management is to reduce stone formation by increasing cystine solubility in urine, which includes lowering the urinary cystine level below its solubility limit. Treatment usually involves alkalinization of the urine and often requires initiating pharmacotherapy with a cystine-binding thiol drug (CBTD) such as tiopronin; however, proper dose adjustment requires accurate measurement of urinary cystine. The goal of this study was to validate a novel high-performance liquid chromatography tandem mass spectrometry (HPLC–MS/MS) method for quantification of cystine in the urine of patients with cystinuria receiving a CBTD. Urine samples were collected over 24 h from 24 patients and separated into 2 aliquots. Chromatographic separation of samples was conducted and separation of cystine from the cysteine-tiopronin drug complex was complete in < 3 min. The method was validated for accuracy, precision, linearity, limit of detection (LOD), and limit of quantification (LOQ). Mean accuracy range was 97.7–102.3%; intermediate precision was high with relative percent difference values calculated at 1.2–9.3%; the calibration curve resulted in a linear response throughout the concentration range (*R*^2^ = 0.998); and the LOD and LOQ were 0.002 and 0.005 mg/mL, respectively. Mean (range) cystine concentrations measured were 111.10 (51.31–179.46) and 242.21 (61.14–741.80) g/L in Aliquots A and B, respectively. The HPLC–MS/MS method presented here indicates that urine cystine can be reliably quantified in patients receiving a CBTD.

## Introduction

Cystinuria is a rare genetic disorder of disrupted resorption of cystine and dibasic amino acids in the proximal tubule of the kidneys [1]. The resulting elevated urinary cystine levels lead to the development of recurrent kidney and bladder stones [2]. Recurrent stones adversely affect activities of daily living, emotional and mental health, and quality of life, and in addition, they confer a higher risk of developing chronic kidney disease (CKD), which has been reported in as many as 70% of patients with cystinuria [3–9].

The goal of cystinuria management is to increase cystine solubility. One contributor to this goal is reduction of the cystine concentration in the urine to below 250 mg/L (its solubility at a urinary pH of 7.0), which is the generally accepted solubility limit in patients to avoid stone formation. Urinary pH is an important determinant of cystine solubility, and solubility increases with increasing alkalinization (750 mg/L at pH 8.0) [7, 10]. Initial conservative management includes adequate fluid intake, dietary restrictions, and urinary alkalinization therapy [10, 11]. If these first-line therapies are not effective, or if a patient has a large, recurrent stone burden, the American Urological Association Guidelines recommend the use of cystine-binding thiol drugs (CBTDs) such as alpha-mercaptopropionyl glycine (tiopronin) [12]. Patients on CBTDs should be monitored through 24-h urine collections, and doses of CBTDs should be adjusted to the lowest effective dose that maintains urinary cystine levels < 250 mg/L [10].

Collection of the 24-h urine sample for evaluation of urine chemistry is the cornerstone of preventive therapy and is recommended for all cystine stone formers. Expert consensus recommendations suggest obtaining a 24-h urine sample 1–2 months after initiating dietary modification or pharmacotherapy. Additional urine samples should be collected periodically to assess changes produced by the therapy, with the goal of increasing solubility and reducing the urinary cystine concentration to < 250 mg/L at urinary pH > 7.0. Clinicians should also evaluate patient adherence as part of the follow-up plan [10]. However, management is not straightforward, as most cystine assays cannot reliably differentiate free cystine from the cysteine-thiol drug complexes in patients taking CBTDs [13, 14]. A cystine capacity test can provide reliable measurements but is impractical for routine clinical practice because of complexity and limited availability [14, 15]. Therefore, a valid, reliable, practical test is needed to quantify urinary cystine levels for patients on CBTDs, guide clinical decision-making, and minimize the risk of adverse events.

Previous methods used to quantify urinary cystine include solid-phase assays, colorimetric reactions, and chromatographic techniques, but these methods are limited in their ability to reliably distinguish cystine from thiol drug-cysteine complexes, complicating clinical interpretation of test results [14, 16, 17]. In a prior study, high‐performance liquid chromatography–tandem mass spectrometry (HPLC–MS/MS) was used to quantify cystine in conditionally immortalized human proximal tubular epithelial cells (ciPTEC) [18]. This fast and reliable assay shares similarities with the method described in this manuscript but analyzed cystine recovered from whole kidney cells instead of urine [18]. The goal of this study was to validate a novel HPLC–MS/MS) method for quantification of cystine in the urine of patients with cystinuria receiving CBTDs.

## Methods

### Materials and equipment

The following chemicals, reagents, and instruments were used: primary HPLC–MS/MS instrumentation (Agilent Technologies, Inc); polar column (Phenomenex, Inc.); analytical balance (Shimadzu Corporation); vortex mixer (Thermo Fisher Scientific); centrifuge (Restek Corporation); pipettors (Globe Scientific); high-performance liquid chromatography (HPLC)–grade water, acetonitrile, isopropanol, and methanol (Honeywell International, Inc.); formic acid and ammonium formate (Supelco); autosampler vials (Shimadzu Corporation); L-cystine (Sigma-Aldrich); R-Cysteine-R-Tiopronin (Travere Therapeutics, Inc.); DL-Cystine-2,2′,3,3,3′,3′-d6 (C/D/N Isotopes Inc.); and cotinine-d3 0.1 mg/mL in methanol (NGX).

### Sample collection

As specified in the 24-h urine collection instructions, patients added 1 g thymol powder and 50 mg gentamicin powder as preservatives to the 24-h urine container before the first urine sample collection. Patients then discarded the first morning urine and began collecting specimens throughout the following 24 h. The final urine collection was conducted the next morning, and total urine volume was recorded by the patient. Approximately 100 mL of urine was transferred from the 4-L 24-h urine collection container to a sample urine cup labeled “A”. The remaining urine sample in the 24-h urine container was mixed with 7 g of sodium carbonate and shaken well. Following incubation for 30 min, the container was shaken again, and approximately 100 mL was poured into a sample urine cup labeled “B”. The samples were shipped overnight to a laboratory at room temperature and were then frozen and sent to the investigators.

The internal standard used for quantification of cystine was cystine-d6. Because no isotopic analogue was available for cysteine-tiopronin, cotinine-d3 was chosen as the surrogate internal standard due to its similar and unique properties. The internal standard was prepared as follows: 100 mg of cystine-d6 was dissolved into 100 mL of 1 N HCl (aq.) to produce a 1.0 mg/mL solution of cystine-d6. A 10 × dilution of the commercially available 0.1 mg/mL solution of cotinine-d3 was prepared in 0.1 N HCl (aq.) to create a 0.01 mg/mL solution of cotinine-d3. Equal parts of the cystine-d6 and cotinine-d3 solutions were mixed to generate the internal standards solution.

A “dilute-and-shoot” HPLC–MS/MS method was utilized, wherein 20 µL of sample, calibrator, or quality control sample was added to a 2 mL HPLC vial. Next, 1400 µL of 0.1 N HCl (aq.) was added, followed by 80 µL of the internal standard mixture.

### HPLC–MS/MS method

Mass spectrometry was conducted using an Agilent 6410 Triple Quadrupole HPLC–MS/MS system (Agilent Technologies, Inc.). HPLC was performed by injecting 10 µL samples into a C18 Polar Column (Phenomenex Luna^®^ Omega 5 µm 100Å50 × 4.6 mm) and separated by an Agilent 1200 series binary pump system (Agilent Technologies, Inc.). Chromatographic separation was performed at a flow rate of 0.400 mL/min using a gradient elution program over 6.5 min. Eluents were 0.1% formic acid in water and 0.1% formic acid in acetonitrile.

Instrument control, data acquisition, and data processing were all performed using the Agilent MassHunter (v.7) software platform (Agilent Technologies, Inc.). Samples were introduced via a positive electrospray ionization with the following source conditions: gas temperature of 300 ºC, gas flow rate of 10 L/min, nebulizer pressure of 15 psi, and capillary voltage of 3500 V. After optimization, two multiple reaction monitoring transitions were selected for cystine and the cysteine-tiopronin complex, whereas 1 was selected for each internal standard. Spectra were scanned over the mass/charge number (m/z) range of 74.1–152.0 atomic mass units and were generated by collision-induced dissociation between 8 and 36 V.

The aim of optimizing the HPLC–MS/MS method was to achieve analyte separation and detection within 3 min. The method was developed in accordance with Food and Drug Administration (FDA) Bioanalytical Method Validation Guidance for Industry.

### Cystine quantification

#### Calibration, linearity, and range

A 6-point calibration curve with target concentrations ranging from 0.03 to 1.00 mg/mL was prepared. Limits of detection (LOD) and limits of quantification (LOQ) were calculated using the standard deviation (SD) of the *y*-intercepts of the calibration curve. A regression analysis with 99% confidence level, which included determination of SD, was performed using the Microsoft Excel Data Analysis ToolPak add-in (Microsoft Corporation, Inc.). LOD was calculated as 3 SDs and LOQ as 10 SDs.

#### Accuracy and precision

Accuracy and repeatability were determined using six replicates of each target concentration calibrator. Accuracy was assessed using mean accuracy percentage, while repeatability was assessed using the calculated SD and the coefficient of variation (CV). Intermediate precision of the current HPLC–MS/MS method was investigated using 3 sets of calibrators prepared over 3 days by different chemists. The second and third set of calibrators were run as controls against the first set. Intermediate precision was assessed using relative percent difference (RPD).

#### Assessment of potential interferences and matrix effects

The susceptibility of the current method to interference from exogenous compounds was assessed using 9 mixtures containing commonly used over-the-counter, prescription, and illicit drugs. Susceptibility to interference from endogenous compounds was evaluated by 15 collections of 24-h urine samples from individuals without cystinuria. All samples were run through the HPLC–MS/MS process and tested for the occurrence of false positives.

Although the use of cystine-D6 as an internal standard was expected to reduce the possibility of signal enhancement or suppression because of matrix effects, testing nevertheless was performed to screen for these potential effects. Cystine solution (2 mg/mL) was added 1:1 (volume:volume) to a sample of blank urine and each of the 9 interference mixtures, and matrix effects were subsequently evaluated.

### Patient samples

The 24-h urine samples taken from 24 patients receiving a CBTD were provided for testing by Travere Therapeutics (San Diego, CA). Upon collection, an aliquot of each patient sample was removed (Cup A), and the remaining specimen was treated with excess sodium carbonate (Cup B) to increase urinary pH and solubilize cystine. All 48 samples were analyzed for cystine using the current HPLC–MS/MS method.

## Results

### HPLC–MS/MS method

**Fig. 1:**
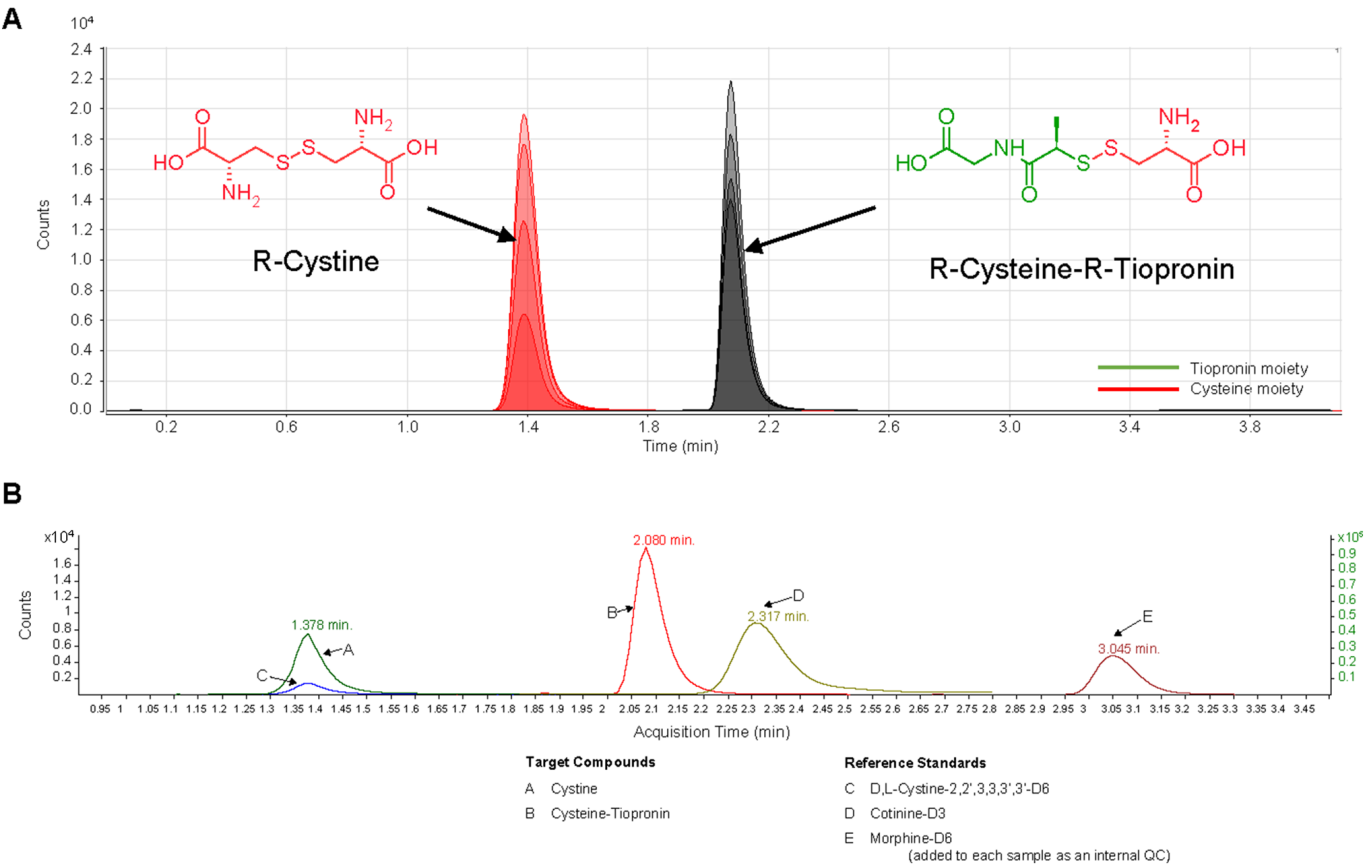
**A** Chromatogram showing separation of cystine from the cysteine-tiopronin complex. R-Cystine, retention time = 1.4 min; R-Cysteine-R-Tiopronin retention time = 2.1 min. **B** Representative chromatogram for cystine and cysteine-tiopronin in a cystinuria patient receiving tiopronin (patient sample 7A)

Chromatographic separation of cystine from the cysteine-tiopronin drug complex was achieved in < 3 min (Fig. 1A).

### Cystine quantification

#### Calibration, linearity, and range

**Fig. 2:**
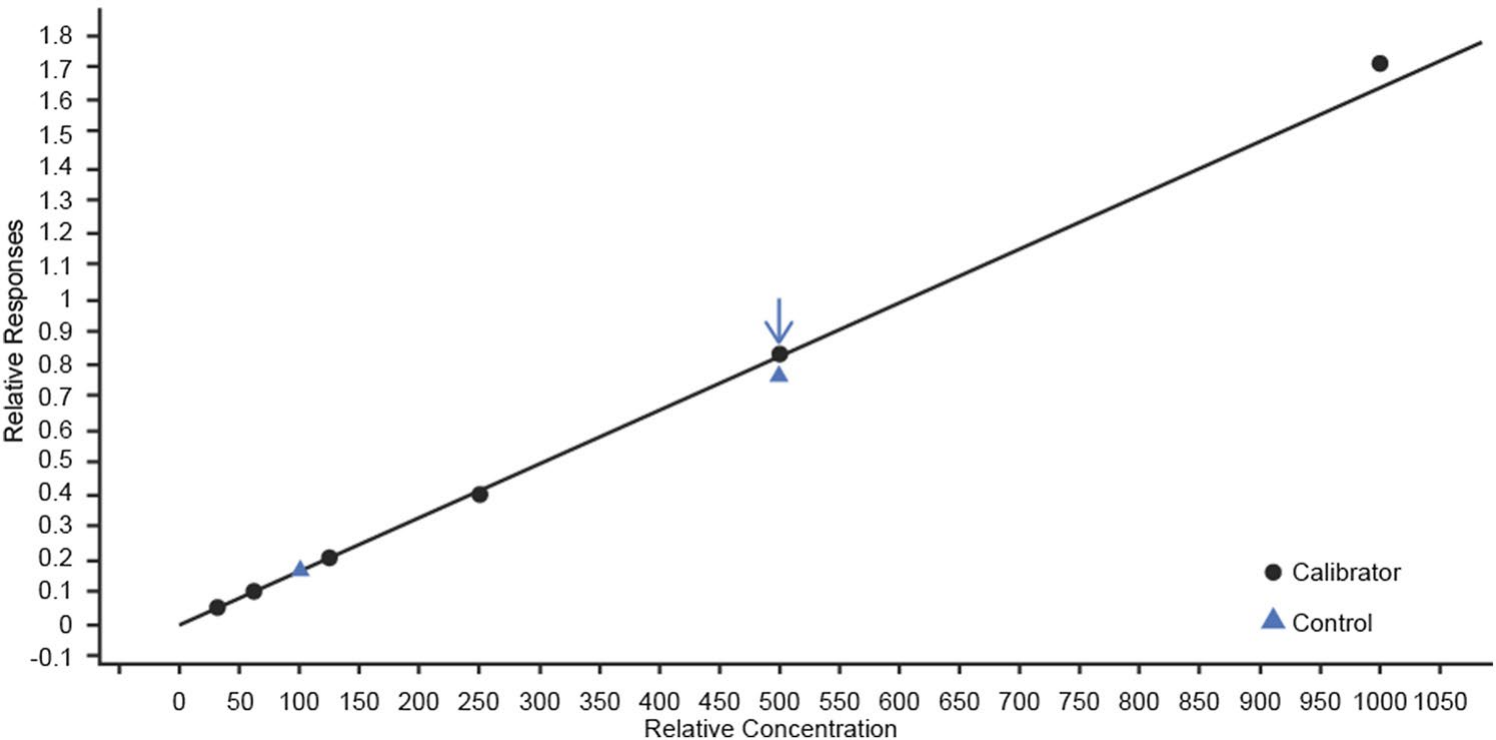
Calibration curve showing a linear response throughout the concentration range for cystine quantification (mg/mL) (*n* = 6). *y* = 0.001637*x* + 2.849557 × 10^–4^; *R*^2^ = 0.99812995

The calibration curve resulted in a linear response throughout the concentration range (*R*^2^ = 0.998; Fig. 2). The LOD and LOQ were 0.002 and 0.005 mg/mL, respectively.

#### Accuracy and precision

Six replicates of calibrators were used to determine accuracy. For each target concentration, mean accuracy ranged from 97.7 to 102.3%, and CVs ranged from 1.2 to 3.3% (Table 1). Precision was defined as a statistical determination of the closeness of agreement between independent test results obtained under specific conditions (the classes of repeatability and intermediate precision apply to the analyses presented here). Assessment of repeatability was conducted using 6 determinations of calibrators, and results were used to calculate the SD and CV of the calibration curve for cystine quantification. The RPD ranged from 1.2 to 9.3%, indicating intermediate precision was high.

**Table 1:** Accuracy and precision for cystine quantification

Sample no.	Target concentration (mg/mL)	Mean calculated concentration (mg/mL), *n* = 6	Mean accuracy (%)	SD (mg/mL)	CV (%)
1	0.0313	0.0315	100.7	0.0005	1.6
2	0.0625	0.0620	99.1	0.0020	3.3
3	0.1250	0.1244	99.5	0.0015	1.2
4	0.2500	0.2443	97.7	0.0058	2.4
5	0.5000	0.5029	100.6	0.0062	1.2
6	1.0000	1.0231	102.3	0.0231	2.3

#### Assessment of potential interferences and matrix effects

No false positives were observed for exogenous or endogenous compounds. A significant matrix effect was observed in 1 interference mixture, which contained (-)-cotinine, (-)-nicotine, acetaminophen, caffeine, ibuprofen, naproxen, phentermine, and *R*,*R*(-)-pseudoephedrine.

### Patient samples

Mean cystine concentrations were 111.10 g/L (range 51.31–179.46 g/L) and 242.21 g/L (range 61.14–741.80 g/L) in Aliquots A and Aliquots B, respectively (Fig. 1B; Table 2).

**Table 2:** Measurement of cystine in 24-h urine samples of patients with cystinuria receiving a CBTD

Sample no.	Measured concentration of cystine (g/L)	
	Aliquot A	Aliquot B^a^
1	128.48	222.62
2	135.72	147.58
3	130.88	741.80
4	112.82	144.84
5	101.36	120.29
6	145.64	196.47
7	139.06	227.47
8	121.87	312.05
9	57.91	186.92
10	112.42	134.02
11	82.35	90.51
12	69.51	122.07
13	138.96	551.10
14	179.46	333.23
15	99.53	181.15
16	61.42	64.82
17	56.00	71.38
18	122.77	242.63
19	143.43	254.35
20	128.67	650.22
21	51.31	61.14
22	119.87	464.59
23	110.98	150.59
24	116.07	141.07
Mean	111.10	242.21

^a^Aliquot B was an alkalinized portion of the 24-h urine collection

## Discussion

Urine supersaturation is strongly correlated with stone mineral composition, and treatments designed for stone prevention decrease supersaturation values [19]. Strategies to prevent stone formation either reduce the absolute amount of cystine or increase its solubility in urine [20]. Medical therapies for cystine stone prevention include increasing fluid intake to 4 L or more per day, limiting sodium consumption to ≤ 2300 mg/day, ingesting a diet low in animal protein, and utilizing potassium citrate therapy to increase urinary pH to 7.0 [11, 12, 21–23]. If conservative management fails, treatment with CBTDs should be initiated, and patients should be monitored periodically with laboratory testing of blood and urine for potential adverse effects, metabolic response, and adherence to prescribed regimens [12]. Adverse events observed during tiopronin therapy include gastrointestinal and dermatological effects, elevations in liver enzymes, and hematologic abnormalities, such as anemia [12, 24]. In addition to monitoring urinary cystine output, analysis of urine chemistries is essential for effective patient care. The composition of urinary analytes can guide treatment choices and provide feedback on treatment efficacy over time. Monitoring changes in liver enzymes, urinary protein excretion, and complete blood cell count can allow the clinician to adjust therapy as needed [20, 22].

In the management of patients with cystinuria, monitoring cystine in the urine can assist clinicians in assessing the efficacy of therapy [11]. Proper adjustment of CBTD dosing is based on the quantification of total cystine in the urine, but methods to measure cystine and cystine solubility have limitations. Historically, urinary cystine measurement has been inaccurate, as quantification methods for routine testing have either been too complex or lack discernment in distinguishing cystine from CBTD–cysteine disulfide compounds. Colorimetric reactions used to measure free sulfhydryl groups in many cystine assays are unable to distinguish cystine from soluble cysteine-thiol drug complexes (formed by CBTDs), so these cystine measurements are unreliable [25]. Although the HPLC technique is able to distinguish between the two, sample preparation in this method may disrupt the cysteine-thiol drug complex, which also leads to inaccurate measurements [25]. In addition, calculating cystine supersaturation is unreliable because pH levels affect cystine solubility [25, 26]. The Litholink assay offered by the Litholink Corp. (Chicago, IL) for determination of cystine capacity measures the solubility of preformed cystine crystals added to patient urine samples, and the results can show a correlation with stone activity; however, the test lacks sensitivity [11, 27]. The HPLC–MS/MS method reported here provides an alternative to laboratory testing offered by Litholink.

Limitations of the study include the small patient sample size (cystinuria is a rare disease) and lack of assessment of clinical outcome. Methodological limitations may be related to patient collection, freezing and shipping between laboratories, pH adjustment to Cup B with sodium carbonate, and freshness of the 24-h urine samples at the time of analysis.

## Conclusions

The HPLC–MS/MS method described here was validated as an effective technique to reliably quantify urinary cystine in patients receiving CBTDs. Optimal cystine control can help prevent future formation of kidney stones by CBTD dose adjustments made based on these results. Offering patients and providers an improved testing methodology can provide insights into patient response to treatment with the ultimate goal of preventing kidney stones.

## Declarations

### Data sharing statements

The data used for the analyses in this manuscript are available on request from Travere Therapeutics, Inc., San Diego, CA.
